# Evaluating the nutrient and fatty acid profiles of black soldier fly larvae (*Hermetia illucens*) raised on various diets in Thailand

**DOI:** 10.3389/finsc.2025.1692096

**Published:** 2026-01-15

**Authors:** Sarayut Pittarate, Chaiwat Arjin, Perumal Vivekanandhan, Kannan Swathy, Chun-I Chiu, Supamit Mekchay, Patipan Hnokaew, Apinya Sartsook, Thanandon Siripan, Korawan Sringarm, Patcharin Krutmuang

**Affiliations:** 1 Office of Research Administration, Chiang Mai University, Chiang Mai, Thailand; 2 Department of Entomology and Plant Pathology, Faculty of Agriculture, Chiang Mai University, Chiang Mai, Thailand; 3 Center of Excellence on Agricultural Biotechnology: (AG-BIO/MHESI), Bangkok, Thailand; 4 Center of Omics for High-Value Agriculture (AgOmics-CMU), Faculty of Agriculture, Chiang Mai University, Chiang Mai, Thailand; 5 Department of Animal and Aquatic Sciences, Faculty of Agriculture, Chiang Mai University, Chiang Mai, Thailand

**Keywords:** black soldier fly larvae, fatty acid profile, nutritional value, *Hermetia illucens*, zero hunger, responsible consumption and production, life on land

## Abstract

**Introduction:**

Black soldier fly larvae (BSFL) have gained increasing attention as a sustainable alternative protein source for animal feed, particularly when reared on organic by-products. This study evaluated the nutritional composition of BSFL reared on different organic substrates to support sustainable feed production.

**Methods:**

BSFL were reared on five organic substrates: chicken feed, pig feed, soy milk residue, coconut press cake, and perilla cake. Larvae were cultivated in metal trays (23 × 15 cm) for 2–4 weeks under controlled conditions (28 ± 2 °C; 65 ± 5% relative humidity). At the prepupal stage, larvae were harvested and analyzed for dry matter, crude protein, crude fiber, ether extract, ash, growth performance, and fatty acid profiles.

**Results:**

Significant differences in nutritional composition were observed among substrates. Crude protein content was high in larvae fed chicken feed (50.55 ± 0.07%), pig feed (52.10 ± 0.14%), soy milk residue (52.15 ± 0.78%), and perilla cake (47.20 ± 0.00%). Crude fiber was highest in larvae fed soy milk residue (7.19 ± 1.48%) and perilla cake (5.38 ± 0.25%). Fatty acid analysis revealed substantial levels of saturated and unsaturated fatty acids, including palmitic, oleic, and linoleic acids. Larvae reared on coconut press cake showed the highest saturated fatty acid content (74.91 ± 0.03%), while those fed soy milk residue exhibited the highest oleic (26.68 ± 0.06%) and linoleic acid (38.44 ± 0.07%) contents, resulting in increased polyunsaturated fatty acids (38.57 ± 0.03%).

**Discussion:**

The findings demonstrate that organic by-products commonly available in Thailand are suitable substrates for BSFL production and significantly influence larval nutritional quality. These substrates enable the production of nutrient-rich, cost-effective, and sustainable insect-based feed, contributing to responsible consumption, waste valorization, and food security.

## 1 Introduction

Global food demand is expected to rise by 70% by 2050 to meet the needs of the estimated 9.7 billion people who are expected to inhabit the planet by that time (1). Significant dietary shifts are already underway, with a growing preference for animal-based foods—particularly milk, meat, fish, and eggs—and these trends are expected to continue (2). Economic growth, rapid migration from rural to urban areas, and increased awareness of nutritional needs have all accelerated changes in dietary patterns. Cereal and meat production are expected to increase from 2.1 billion and 258 million tons produced per year between 2005 and 2007 to 3.0 billion and 455 million tons, respectively, by the middle of the current century, raising global concerns about food security (3).

In the developing world, the livestock sector has significant potential to alleviate poverty and improve food security (4). Poultry farming in Thailand is a significant source of income, particularly in rural areas, contributing over 25% to the agricultural gross domestic product (GDP) and approximately 5%–6% to the nation’s overall GDP. However, feed costs account for more than 70% of production costs, highlighting the critical role that economic feeds and their availability can play in successful poultry farming (5, 6). Feed constituents suitable for direct human consumption, such as soybeans and fish, are expensive due to food–feed competition and collectively raise feed costs (6). Furthermore, because of overfishing, global catches from marine fish stocks have decreased over time (7), increasing the price of fishmeal, which is not only used to feed livestock but also a major protein source in farmed fish feed (3, 8, 9). Moreover, the expansion of soybean cultivation, especially in tropical regions, has led to land grabbing, deforestation, and a range of adverse social and environmental impacts.

Because several insect species can feed on various types of organic waste streams, large-scale insect rearing presents a promising and innovative alternative for sustainable food and feed production (10). Furthermore, insects are valuable reservoirs of proteins, fatty acids, micronutrients, and energy (11–13). In general, insect proteins have a favorable amino acid profile, including essential amino acids that are often limiting in plant-based protein sources for non-ruminants, such as lysine, threonine, and methionine (10, 14–16).

Among the insect species identified as promising alternative protein sources for animal feed are the black soldier fly (BSF), *Hermetia illucens* L. (Diptera: Stratiomyidae); the common house fly, *Musca domestica* L. (Diptera: Muscidae); and the yellow mealworm, *Tenebrio molitor* L. (Coleoptera: Tenebrionidae) (17). The initial two larval instars typically develop in animal manure but can also thrive on a wide range of organic waste substrates, including coffee pulp, vegetable residues, food service waste, municipal organic matter, straw, dried distillers’ grains with solubles (DDGS), and fish waste. These larvae can effectively reduce the volume of organic waste by 50%–60%, converting it into high-protein biomass (10, 18).

Black soldier fly larvae (BSFL) have a dry weight containing up to 50% crude protein (CP), up to 35% lipids, and an amino acid profile similar to that of fishmeal (19). They are recognized and used as alternative protein sources for poultry, pigs, and several species of fish and shrimp (13). The adult fly can live for 1 to 2 weeks without feeding, as it appears to rely on fat body reserves acquired during the larval stages, and it can even survive and live longer when fed with water (3, 10). It does not transmit diseases, and actively feeding BSFL secrete an info-chemical that repels other flies, thereby repelling potential insect pests and disease vectors such as *M. domestica* (20). BSFL larvae have a significant influence on the reduction of *Escherichia coli* and *Salmonella enterica* population in cow dung (20). Similarly, Thornton (21) reported the same effect on *E. coli* in chicken manure.

The economic viability of utilizing insects as animal feed largely depends on the availability and affordability of organic waste streams in both developed and developing countries. However, limited research has comprehensively evaluated the nutritional quality of BSFL reared on diets formulated under the resource constraints typical of developing regions. Unlike controlled, rationed diets, organic waste streams in such contexts exhibit considerable variability in nutritional composition and may contain contaminants, including heavy metals. Therefore, the present study aimed to perform a holistic comparative analysis of the nutritional profile of BSFL reared on commonly available organic food wastes found in urban areas of Thailand and similar settings in the developing world. Such comparative evaluations are essential to identify the most suitable organic substrates for industrial-scale BSFL production in Thailand.

## 2 Material and methods

### 2.1 Research area

The research was conducted at Chiang Mai University, within the Department of Entomology and Plant Pathology and the Department of Animal and Aquatic Science in Thailand.

### 2.2 Insect rearing, maintenance, and feeds selection

The adult BSFL insect population that serves as the stock colony was originally maintained and reared at Chiang Mai University’s Department of Entomology and Plant Pathology in Thailand. Adult BSFs are maintained in an outdoor metal-framed cage measuring 1.81 × 1.81 × 1.81 m, fitted with a 1.5-mm mesh screen that allows ample exposure to natural daylight. The temperature is regulated at 27 ± 3 °C to promote mating activity. Water mixed with a 10% honey solution is provided to enhance adult longevity. To stimulate oviposition, corrugated cardboard and substrate materials (SM) are placed inside the cage as oviposition sites. BSF prepupae used for analyses were obtained from a series of experiments carried out in the Insect Pathology Laboratory. Diets selection, rearing substrates design, and larvae rearing experiments were managed by Chiang Mai University’s Department of Entomology and Plant Pathology (Thailand). BSFL were reared under controlled conditions [27 ± 1 °C, 60%–70% relative humidity (RH)] on various diets; details of the chemical composition of the substrates are reported in **Table 1**. Five treatments with three replications each were conducted, releasing exactly 20 g of BSFL (second and third instar) per substrate. The rearing experiments lasted up to 24 days, with regular monitoring of prepupae development. At each control, the prepupae were collected, killed by freezing, stored at −20 °C for 24 h, then freeze-dried and kept at −20 °C until further analysis.

**Table 1:** Chemical composition of diets for feed BSFL.

Chemical composition (%)	Chicken feed	Pig feed	Soy milk residue	Coconut press cake	Perilla cake	SEM	*p*-value
Dry matter	89.93 ± 0.16^a^	95.21 ± 6.75^a^	90.52 ± 0.37^a^	94.78 ± 0.04^a^	90.21 ± 0.02^a^	1.06	0.327
Crude protein	23.85 ± 0.07^b^	23.30 ± 0.85^b^	24.65 ± 0.07^b^	7.61 ± 0.21^a^	29.81 ± 0.05^c^	2.50	0.000
Crude fiber	6.23 ± 0.25^a^	5.14 ± 0.12^a^	11.27 ± 0.2^b^	30.78 ± 2.14^c^	30.50 ± 0.34^c^	3.84	0.000
Ether extract	3.44 ± 0.54^a^	9.50 ± 0.03^b^	10.20 ± 1.20^b^	27.28 ± 0.12^c^	10.52 ± 0.04^b^	2.66	0.000
Ash	5.23 ± 0.30^c^	7.13 ± 0.14^d^	2.99 ± 0.47^b^	0.86 ± 0.03^a^	5.23 ± 0.04^c^	7.21	0.000

^*a,b,c,d^ Different letters within the same row indicate a statistically significant difference (*p* < 0.05).

### 2.3 Chemical composition of the diets and BSFL

The chicken feed, pig feed, soy milk residue, coconut press cake, and perilla cake were purchased from a Chiang Mai, Thailand, local market. The chemical composition of these five diets (chicken feed, pig feed, soy milk residue, coconut press cake, and perilla cake) was examined in the current study and is presented in **Table 1**. The chemical composition of diet and BSFL was analyzed according to the method of AOAC International (22). Dry matter (DM) was measured by 5 h of oven-drying at 95–100 °C (method 934.01). Following the complete combustion of samples in a muffle furnace at 600 °C for 2 h, the ash content was calculated (method 942.05). The macro-Kjeldahl method (method 2001.11) was used to determine the CP content, which was calculated as nitrogen 6.25. Dichloromethane was used as the solvent in a Soxhlet extraction to extract the ether extract (EE) for 16 h (method 920.39), and crude fiber (CF) was determined using method 962.09.

### 2.4 Fatty acid profile of BSFL

The fatty acid profile was analyzed following the method described by Sringarm et al. (23) with minor modifications. Lipids were extracted from the samples using the Soxhlet extraction technique (method 920.39). Fatty acid methyl esters (FAME) were prepared according to the procedure outlined by Morrison and Smith (1964). Analysis was conducted using a Shimadzu GC-2030 gas chromatograph equipped with a flame ionization detector (FID) (Kyoto, Japan). Separation was achieved on a wall-coated fused silica capillary column (RT-2560, RESTEK, Bellefonte, PA, USA) measuring 0.25 mm × 100 m × 0.25 μm, with helium as the carrier gas at a constant flow rate of 1 mL/min. The injector temperature was maintained at 250 °C. The oven temperature program began at 50°C, ramping to 220 °C at 10 °C/min, held for 35 min, followed by an increase to 230 °C at a rate of 5 °C/min, maintained for 20 min. The FID temperature was set at 250 °C, and an injection volume of 1 μL was used. Fatty acids were identified by comparing retention times with those of standard compounds. The fatty acid composition was expressed as a percentage of the total identified fatty acids. Additionally, the fatty acid profiles of the five diet formulations are summarized in **Table 2**.

**Table 2:** Fatty acid composition of experimental diets before feed.

Fatty acid (g/100 g fat)	Formula	Chicken feed	Pig feed	Soy milk residue	Coconut press cake	Perilla cake	SEM	*p*-value
Lauric acid	C12:0	0.26 ± 0.01^a*^	5.19 ± 0.00^a^	2.99 ± 3.96^a^	51.48 ± 0.00^b^	Nd^**^	6.627	0.000
Myristic acid	C14:0	7.24 ± 0.02^b^	2.35 ± 0.02^a^	3.82 ± 2.75^ab^	20.94 ± 0.00^c^	0.01 ± 0.00^a^	2.489	0.000
Pentadecylic acid	C15:0	2.43 ± 0.01^b^	Nd	0.93 ± 1.32^ab^	0.01 ± 0.00^a^	0.01 ± 0.01^a^	0.345	0.033
Palmitic acid	C16:0	17.72 ± 0.03^d^	18.04 ± 0.01^e^	15.60 ± 0.00^c^	11.34 ± 0.00^b^	6.90 ± 0.00^a^	1.416	0.000
Palmitoleic acid	C16:1	0.66 ± 0.00^d^	0.16 ± 0.00^b^	0.47 ± 0.03^c^	0.03 ± 0.00^a^	0.19 ± 0.03^b^	0.077	0.000
Heptadecanoic acid	C17:1	0.19 ± 0.12^a^	0.03 ± 0.00^a^	0.11 ± 0.06^a^	0.01 ± 0.00^a^	0.01 ± 0.00^a^	0.027	0.094
Stearic acid	C18:0	4.01 ± 0.03^d^	2.78 ± 0.00^a^	4.85 ± 0.02^e^	3.23 ± 0.00^c^	3.15 ± 0.01^b^	0.248	0.000
Elaidic acid	C18:1n9t	0.11 ± 0.01^b^	0.29 ± 0.00^c^	0.25 ± 0.02^c^	0.04 ± 0.00^a^	Nd	0.037	0.000
Oleic acid	C18:1n9c	37.71 ± 0.05^d^	34.54 ± 0.01^e^	24.69 ± 0.13^c^	9.72 ± 0.00^a^	12.45 ± 0.01^b^	3.330	0.000
Linoleic acid	C18:2n6c	29.94 ± 0.07^c^	33.21 ± 0.01^d^	40.23 ± 0.49^e^	3.02 ± 0.00^a^	20.74 ± 0.01^b^	4.280	0.000
Arachidic acid	C20:0	0.11 ± 0.00^a^	1.00 ± 0.01^a^	0.47 ± 0.63^a^	0.04 ± 0.00^a^	0.20 ± 0.00^a^	0.135	0.082
α-Linolenic acid	C18:3n3	0.16 ± 0.04^ab^	0.40 ± 0.00^c^	0.021 ± 0.06^b^	0.07 ± 0.00^a^	56.29 ± 0.01^d^	7.477	0.000
Heneicosylic acid	C21:0	5.37 ± 0.01^d^	1.62 ± 0.01^b^	5.22 ± 0.05^c^	0.07 ± 0.00^a^	Nd	0.796	0.000
	SFA	37.14 ± 0.08^d^	30.98 ± 0.03^b^	33.88 ± 0.49^c^	87.12 ± 0.00^e^	10.26 ± 0.01^a^	8.474	0.000
	PUFA	30.20 ± 0.02^b^	33.61 ± 0.01^c^	40.44 ± 0.56^d^	3.09 ± 0.00^a^	77.00 ± 0.00^e^	7.918	0.000
	MUFA	32.67 ± 0.06^d^	35.01 ± 0.01^e^	25.51 ± 0.06^c^	9.79 ± 0.00^b^	12.65 ± 0.02^a^	3.418	0.000
	PUFA: MUFA	0.92 ± 0.00^a^	0.96 ± 0.00^a^	1.09 ± 0.73^a^	0.32 ± 0.00^a^	6.09 ± 0.01^b^	0.712	0.000
	SFA: PUFA	1.23 ± 0.00^d^	0.92 ± 0.00^c^	0.84 ± 0.02^b^	28.89 ± 0.00^e^	0.13 ± 0.00^a^	3.662	0.000
	SFA: MUFA	1.14 ± 0.01^c^	0.89 ± 0.01^b^	1.33 ± 0.01^d^	8.90 ± 0.00^e^	0.81 ± 0.00^a^	1.051	0.000

^*a,b,c,d,e^ Different letters within the same row indicate a statistically significant difference (*p* < 0.05), ^**^ Nd means not detected.

### 2.5 Statistical analysis

Statistical analyses were performed using the Statistical Package for the Social Sciences (SPSS, version 16.0). Data normality was assessed using Levene’s test for homogeneity of variances. Subsequently, a one-way analysis of variance (ANOVA) was conducted employing the general linear model (GLM) procedure to evaluate significant differences among group means. When significant *F*-values were observed, Tukey’s *post-hoc* test was applied to separate means at a significance level of *p* < 0.05.

## 3 Results

### 3.1 The productive performance of BSFL

**Figure 1:**
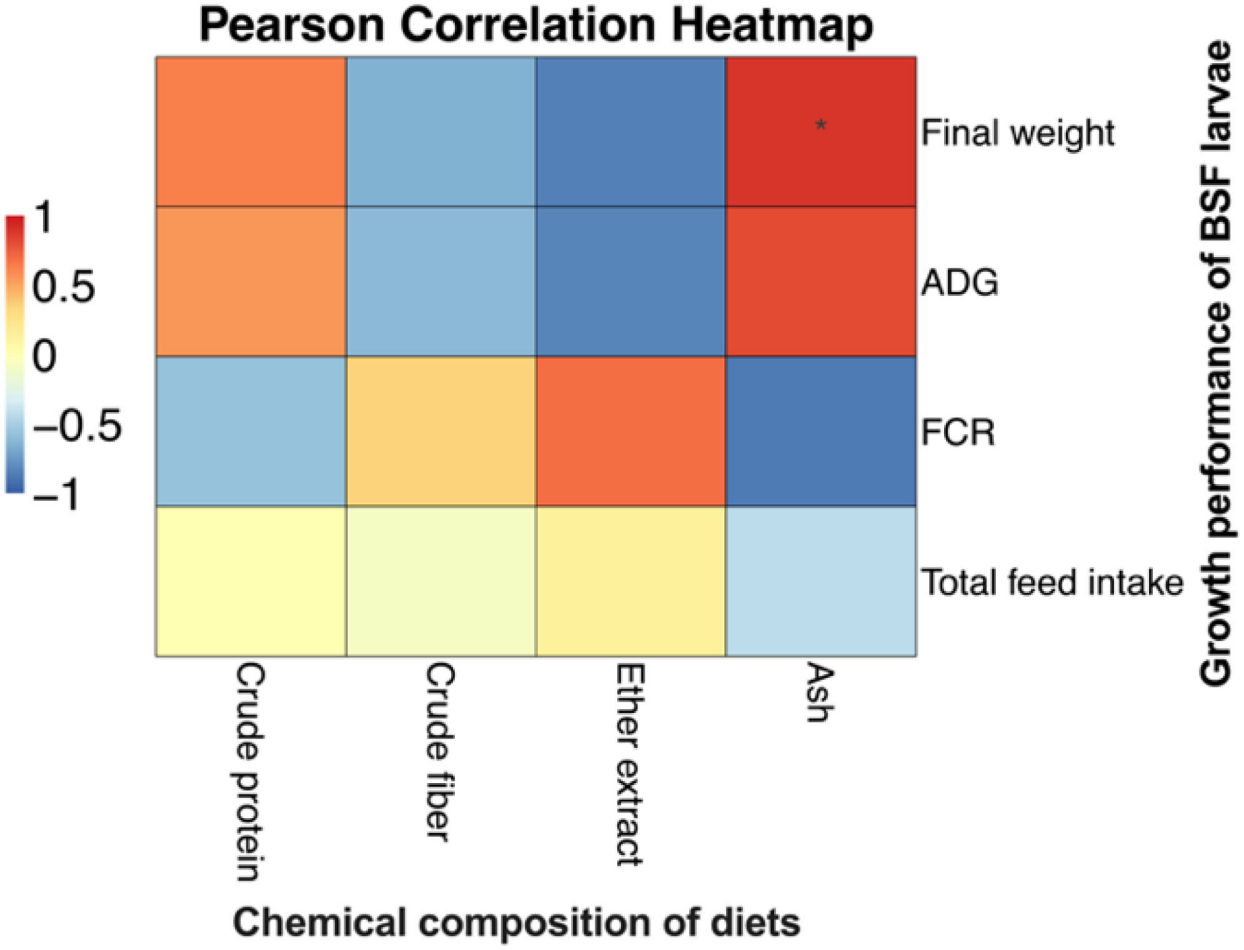
The Pearson correlation between dietary chemical composition and BSFL growth performance, An asterisk indicates a statistically significant difference (*p < 0.05).

The productive performance of BSFL fed various dietary substrates is presented in **Table 3**. Significant differences (*p* < 0.05) were observed across all performance metrics among the treatments. Larvae raised on chicken feed exhibited the highest final weight and average daily gain (ADG), indicating superior growth efficiency. In contrast, larvae fed soy milk residue and coconut press cake showed elevated feed conversion ratios (FCRs), reflecting less efficient nutrient utilization. Additionally, rearing BSFL on chicken feed and perilla cake provided a highly reliable diet. The costs associated with rearing were also reduced when using perilla cake, chicken feed, and coconut press cake as feed sources. **Figure 1** illustrates the relationship between feed composition and BSFL growth performance. The data demonstrate a positive correlation between higher CP and ash content and the final weight and ADG of BSFL. A negative correlation was observed between FCR and feed intake in diets with high ash content.

**Table 3:** Growth performance of BSFL after feeding diets.

Items	Chicken feed	Pig feed	Soy milk residue	Coconut press cake	Perilla cake	SEM	*p*-value
Initial weight, g	20	20	20	20	20	0.00	–
Final weight, g	1,042.37 ± 39.17^d*^	931.12 ± 67.63^d^	510.00 ± 91.95^b^	337.16 ± 95.04^a^	753.10 ± 39.26^c^	71.41	0.000
ADG, g/day	73.03 ± 2.81^e^	53.60 ± 3.98^d^	23.33 ± 4.38^b^	13.22 ± 3.96^a^	43.18 ± 2.31^c^	5.75	0.000
FCR	1.66 ± 0.07^a^	2.42 ± 0.19^a^	8.16 ± 1.32^b^	7.93 ± 2.11^b^	2.75 ± 0.61^a^	0.80	0.000
Total feed intake, g	1,700.00 ± 0.00^a^	2,200.00 ± 0.00^b^	3,916.67 ± 144.34^c^	2,383.33 ± 202.07^b^	2,000.00 ± 346.41^ab^	210.57	0.000
Feed cost, THB^†^	2.71 ± 0.10^a^	5.26 ± 0.41^c^	3.91 ± 0.60^b^	3.70 ± 0.87^ab^	2.67 ± 0.58^a^	0.28	0.001

†THB, Thai Baht; Feed cost wascalculated per 100 g of larva, ^*a,b,c,d^ Different letters within the same row indicate a statistically significant difference (*p* < 0.05).

### 3.2 Chemical composition of the BSFL after feeding diets

**Figure 2:**
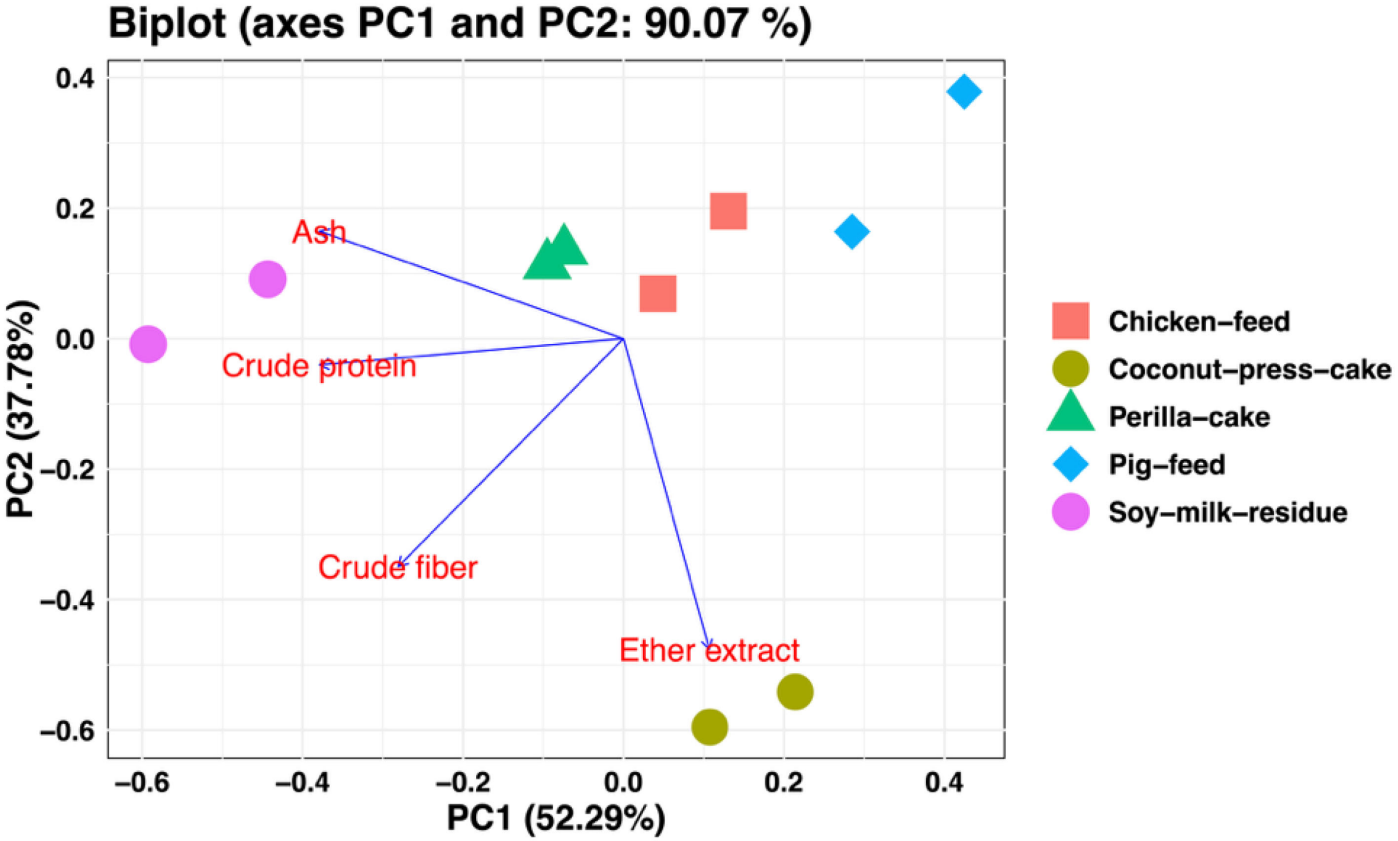
The relationship between the chemical constituents of larvae and their dietary types was illustrated by a principal component analysis (PCA) biplot. PC1 accounted for 52.29% of the total variability along the *X*-axis, while PC2 accounted for 37.78% along the *Y*-axis.

In this study, the dietary supplementation on the chemical composition of the BSFL after feeding five different kinds of diets is shown in **Table 4**. The DM and crude fiber of all diets show no significantdifferences (*p* > 0.05). However, significant differences were observed in the CP, ether extract, and ash contents among the treatments. We found that the CP and ether extract show the highest percentage from larvae fed on soy milk residue and coconut press cake, respectively. Furthermore, we discovered that the chemical components of BSFL were influenced by their diet. The ether extract composition of the larvae that were fed coconut press cake was found to be higher, while the CP composition was lower. The PCA biplot (**Figure 2**) revealed clear differentiation among feed substrates based on their proximate compositions, particularly ether extract and CP contents. Furthermore, these compositional differences were reflected in the biochemical profiles and growth performance of *H. illucens* larvae. Larvae reared on coconut press cake contained a higher proportion of ether extract but lower CP content, whereas those fed chicken feed exhibited the greatest pupal weight, length, and width (data not shown, *p* < 0.05). These findings suggest that lipid-rich diets promote fat accumulation, while protein-rich diets enhance larval development and biomass yield.

**Table 4:** Chemical composition of BSFL after feeding diets.

Chemical composition (%)	Chicken feed	Pig feed	Soy milk residue	Coconut press cake	Perilla cake	SEM	*p*-value
Dry matter	79.66 ± 19.30^a^	96.58 ± 4.58^a^	95.30 ± 0.09^a^	96.86 ± 0.21^a^	93.91 ± 0.07^a^	3.01	0.372
Crude protein	50.55 ± 0.07^c^	52.10 ± 0.14^d^	52.15 ± 0.78^d^	22.05 ± 0.49^a^	47.20 ± 0.00^b^	3.84	0.000
Crude fiber	3.76 ± 1.48^a^	4.03 ± 3.40^a^	7.19 ± 1.48^a^	3.71 ± 0.35^a^	5.38 ± 0.25^a^	0.61	0.361
Ether extract	28.05 ± 0.03^c^	22.35 ± 0.19^b^	17.92 ± 0.39^a^	65.37 ± 0.50^e^	30.68 ± 0.70^d^	5.61	0.000
Ash	7.40 ± 0.02^b^	10.45 ± 0.08^c^	9.98 ± 0.63^c^	3.27 ± 0.16^a^	9.70 ± 0.07^c^	0.89	0.000

^*a,b,c,d,e^ Different letters within the same row indicate a statistically significant difference (*p* < 0.05).

### 3.3 Fatty acid profiles in BSFL

**Figure 3:**
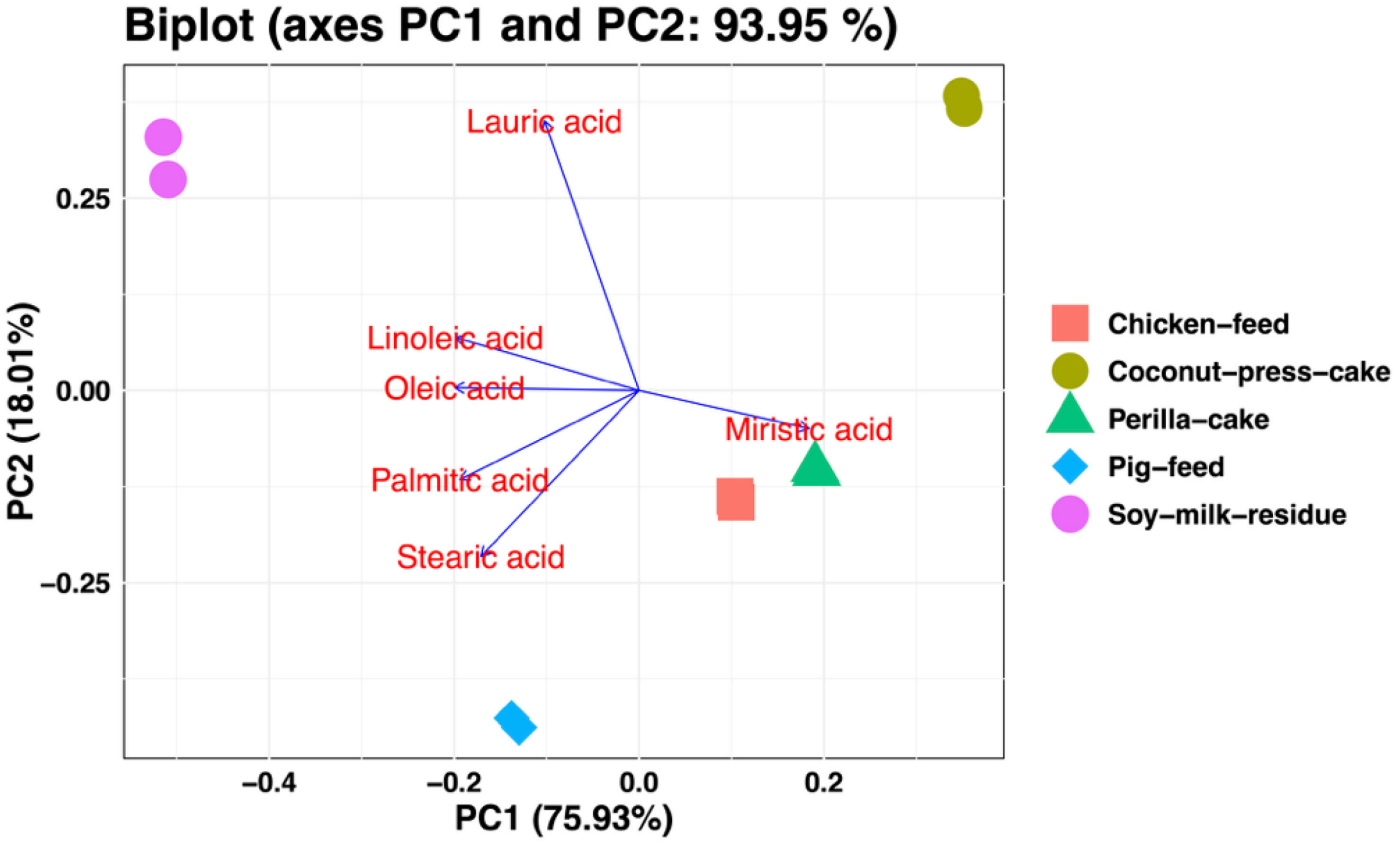
Principal component analysis (PCA) biplots were used to illustrate the correlation between the fatty acids in larvae and their dietary type. In the *X*-axis, PC1 accounted for 75.93% of the total variability, whereas PC2 accounted for 18.01% on the *Y*-axis.

**Figure 4:**
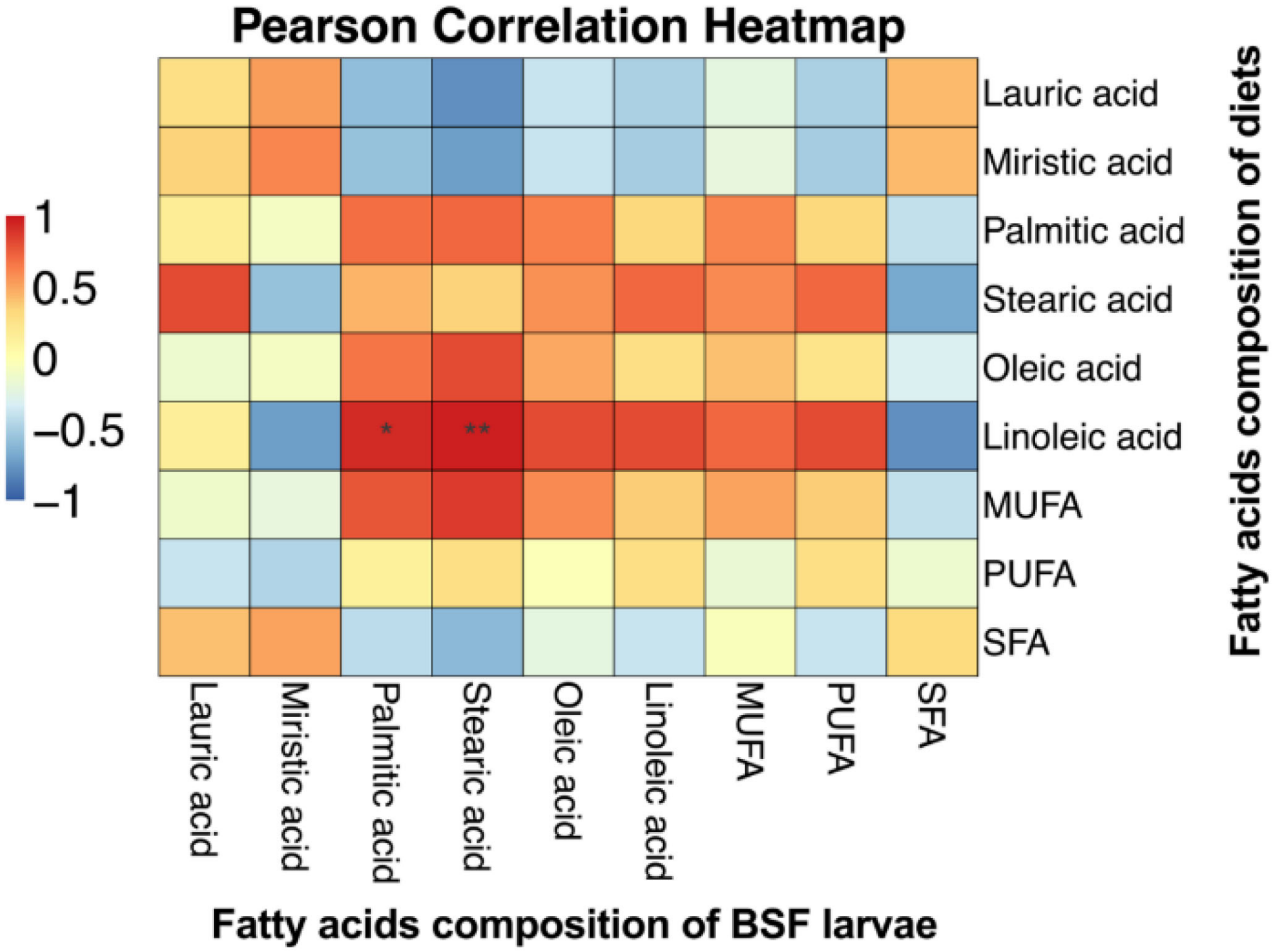
The Pearson correlation between the fatty acid composition of the diet and that of the BSFL, Asterisks indicate statistically significant differences (*p < 0.05, **p < 0.01).

To investigate the impact of diet on the fatty acid composition of growing BSFL, samples were collected at the prepupal stage for fatty acid profile analysis. The results demonstrated that diet significantly influenced the levels of saturated fatty acids (SFAs), monounsaturated fatty acids (MUFAs), and polyunsaturated fatty acids (PUFAs) in the BSFL (*p* < 0.05). However, the heneicosylic acid (C21:0) content was significantly increased in SFAs when fed to BSFL by perilla cake. Moreover, in MUFA and PUFA, oleic acid (C18:1n9c) and linoleic acid (C18:2n6c) from the BSFL feeding on soy milk residue showed higher levels than other diets, while the α-linolenic acid (C18:3n3) in PUFAs showed no significant difference. Furthermore, we found that the ƩSFA, ƩPUFA, and ƩMUFA had significant differences. The ƩSFA of BSFL fed on coconut press cake was higher than other diets. While the larvae fed on soy milk residue showed the highest total fatty acids ƩPUFA and ƩMUFA). Regarding the ratio of fatty acids, the results revealed a significant difference. The ratio of saturated to unsaturated fatty acids (SFA : PUFA) was significantly increased in BSFL fed coconut press cake (**Table 5**). In addition, this study reveals that the various diets resulted in significantly elevated levels of fatty acids in BSFL (*p* < 0.05). Principal component analysis (PCA) showed that dietary substrates had a significant impact on the fatty acid composition of *H. illucens* larvae (**Figure 3**). Dietary sources contributed substantially to the variation in fatty acid profiles, as evidenced by the first two principal components (PC1 = 75.93% and PC2 = 18.01%), which together explained 93.94% of the total variance. Lauric acid (C12:0), a major fatty acid in lipids derived from coconuts, was strongly correlated with the distinct separation of larvae raised on coconut press cake along PC2. Larvae fed chicken feed, on the other hand, clustered with stearic acid (C18:0) and palmitic acid (C16:0), indicating that these diets promoted the buildup of long-chain SFA. In contrast, higher amounts of oleic acid (C18:1n9c) and linoleic acid (C18:2n6c) were linked to larvae raised on soy milk residue and perilla cake, indicating the unsaturated lipid composition of these substrates. These results show that the fatty acid composition of BSFL is directly influenced by dietary lipid profiles through substrate-driven metabolic regulation. Significant relationships between dietary and larval fatty acid compositions were shown by the Pearson correlation heatmap (**Figure 4**). In larvae, dietary linoleic acid exhibited a strong positive correlation with both palmitic and stearic acids, suggesting that it may play a role in the synthesis of new fatty acids. Conversely, there was a negative correlation between dietary lauric acid and unsaturated fatty acids, indicating that larvae raised on coconut-based substrates had a metabolic preference for the accumulation of saturated lipids.

**Table 5:** Fatty acid composition of BSFL after feeding diets.

Fatty acid (g/100 g fat)	Formula	Chicken feed	Pig feed	Soy milk residue	Coconut press cake	Perilla cake	SEM	*p*-value
Lauric acid	C12:0	0.85 ± 0.01^b*^	0.53 ± 0.03^a^	1.72 ± 0.07^d^	1.19 ± 0.00^c^	0.52 ± 0.01^a^	0.15	0.000
Myristic acid	C14:0	34.79 ± 0.03^d^	22.96 ± 0.11^c^	1.40 ± 0.02^a^	38.40 ± 0.00^e^	21.97 ± 0.03^b^	4.40	0.000
Pentadecylic acid	C15:0	9.58 ± 0.00^d^	7.76 ± 0.01^c^	0.20 ± 0.22^a^	19.47 ± 0.01^e^	5.26 ± 0.03^b^	2.12	0.000
Palmitic acid	C16:0	16.40 ± 0.00^c^	18.81 ± 0.05^d^	19.61 ± 0.17^e^	13.65 ± 0.18^a^	14.83 ± 0.01^b^	0.76	0.000
Palmitoleic acid	C16:1	2.04 ± 0.00^c^	1.54 ± 0.04^b^	1.54 ± 0.04^b^	4.25 ± 0.05^d^	1.30 ± 0.00^a^	0.36	0.000
Heptadecanoic acid	C17:1	0.18 ± 0.02^a^	0.63 ± 0.09^b^	0.99 ± 0.05^c^	0.04 ± 0.02^a^	0.60 ± 0.07^b^	0.11	0.000
Stearic acid	C18:0	4.90 ± 0.02^c^	5.92 ± 0.04^e^	5.77 ± 0.04^d^	2.07 ± 0.00^a^	3.68 ± 0.04^b^	0.48	0.000
Elaidic acid	C18:1n9t	0.47 ± 0.00^c^	2.29 ± 0.47^b^	0.73 ± 0.56^d^	0.13 ± 0.00^a^	0.14 ± 0.00^a^	0.08	0.000
Oleic acid	C18:1n9c	18.17 ± 0.00^c^	22.60 ± 0.03^d^	26.68 ± 0.06^e^	15.86 ± 0.03^b^	15.54 ± 0.06^a^	1.43	0.000
Linoleic acid	C18:2n6c	11.24 ± 0.07^b^	17.72 ± 0.10^d^	38.44 ± 0.07^e^	4.61 ± 0.19^a^	12.71 ± 0.01^c^	3.85	0.000
Arachidic acid	C20:0	0.08 ± 0.00^a^	0.36 ± 0.00^b^	0.40 ± 0.04^b^	0.10 ± 0.01^a^	0.10 ± 0.01^a^	0.05	0.000
α-Linolenic acid	C18:3n3	0.08 ± 0.00^a^	0.12 ± 0.02^a^	0.12 ± 0.05^a^	0.04 ± 0.00^a^	0.09 ± 0.00^a^	0.01	0.098
Heneicosylic acid	C21:0	1.21 ± 0.00^c^	0.96 ± 0.00^b^	2.38 ± 0.02^d^	0.04 ± 0.00^a^	23.24 ± 0.07^e^	2.96	0.000
	MUFA	20.86 ± 0.01^c^	25.05 ± 0.11^d^	29.94 ± 0.11^e^	20.28 ± 0.01^b^	17.59 ± 0.12^a^	1.44	0.000
	PUFA	11.33 ± 0.06^b^	17.84 ± 0.11^d^	38.57 ± 0.03^e^	4.81 ± 0.01^a^	12.83 ± 0.05^c^	3.84	0.000
	SFA	67.81 ± 0.06^c^	57.11 ± 0.01^b^	31.51 ± 0.08^a^	74.91 ± 0.03^e^	69.60 ± 0.16^d^	5.16	0.000
	PUFA: MUFA	0.54 ± 0.00^b^	0.72 ± 0.01^c^	1.29 ± 0.01^e^	0.24 ± 0.00^a^	0.73 ± 0.00^d^	0.11	0.000
	SFA: PUFA	5.99 ± 0.04^a^	3.21 ± 0.21^a^	0.82 ± 0.00^a^	15.57 ± 0.06^b^	2.94 ± 3.56^a^	1.77	0.001
	SFA: MUFA	3.25 ± 0.00^c^	2.28 ± 0.01^b^	1.06 ± 0.01^a^	3.70 ± 0.01^d^	3.96 ± 0.04^e^	0.35	0.000

^*a,b,c,d,e^ Different letters within the same row indicate a statistically significant difference (*p* < 0.05).

## 4 Discussion

Currently, the demand for food is driven by the continuous growth of the global population. Consequently, the increase in food demand leads to a corresponding rise in food waste. The composition of the rearing substrates is presented in **Table 1**. The five groups of tested diets differed from one another, primarily due to the plant by-products used: (i) fruit peels and pulp (coconut cake, perilla cake, and soy milk residue) and (ii) animal feed (chicken feed and pig feed), which served as the BSF rearing substrates in the laboratory colony. **Table 1** shows that the different ingredients used in the five groups correspond to varying proximate compositions of the diets. Despite these compositional differences, all diets supported BSF growth, with more than 90% of the initial 20 g of BSFL reaching the prepupal stage in all trials. However, there were consistent variations in the total weight of the final prepupae produced (**Table 3**). The results indicated that BSFL fed coconut press cake exhibited the lowest performance in terms of final weight, ADG, FCR, and feed intake. Analysis of the chemical composition of coconut press cake revealed that ether extract was the most abundant component, which resulted in reduced feed intake by the larvae. This outcome aligns with the findings of previous studies (19, 24), which observed that caterpillars raised on a high-fat diet exhibited reduced growth rates and smaller sizes due to decreased feed intake. Chemical analysis of BSFL showed that only those fed coconut press cake had low protein content, while their ether extract content was higher compared to other diets (17, 19). Moraes et al. (2020) reported that coconut cake contains an ether extract composition of 38.08%, while perilla cake had 10.52% ether extract (25). Coconut press cake, which is a residual product that contains a significant quantity of fat, is typically produced by pressing coconuts to extract coconut milk. It was determined that larvae fed chicken feed, pig feed, soy milk residue, and perilla cake had substantially higher CP levels than the larvae fed coconut cake. It is a scenario constructed because the diets in question contain a higher protein content than coconut cake. The high content of CP in the diet led to improved FCR of larvae. However, this finding differs with the results of Kröncke et al. (26), who reported that a reduced dietary protein content is associated with improved FCR. Additionally, the ADG of larvae that were fed coconut press cake was significantly lower than that of the other group. It is possible that the larvae’s reduced feed consumption is a result of their high-fat diet (27).

The chemical composition of BSFL after feeding on various diets revealed that larvae fed coconut press cake contained the lowest CP compared to other groups. In this study, the coconut press cake diet exhibited the lowest protein level among the diets. According to Fuso et al. (28), the protein content of BSFL is primarily influenced by the fiber and protein content of the diet. The larvae’s high protein diet was associated with the presence of a high-protein feed source. Specifically, the BSFL fat content was strongly affected by dietary nutrient concentration, while larval protein content varies within narrow limits (29). More specifically, our data show that increasing the amount of plant protein in the diet enables BSFL to convert a progressively higher proportion of it into their own animal protein. In fact, while larvae on low-protein diets were able to convert more than 90%, this percentage dropped to 10% for the most protein-rich diets (28, 30). This suggests that the amount of protein in the rearing substrate is crucial for BSFL growth and protein content up to a minimum threshold, beyond which it becomes less significant. These experimental findings align with recent research on digestive enzyme expression and production in BSF (31). Previous studies have identified cellulase genes in the intestinal microflora of BSFL (30, 32). A plausible explanation for the observed relationship is that the ability to utilize more and better fiber biomass enhances larval growth, which, in turn, increases protein production (28). Moreover, this study demonstrated that larvae fed on protein-poor substrates (such as coconut press cake) have higher lipid content and consequently lower protein content (as a percentage of dry mass) (28, 33). Therefore, to obtain prepupae with a high protein content, **Table 4** suggests that rearing them on a substrate containing 20%–23% protein by weight offers a good compromise to maintain an advantageous conversion rate.

Regarding the fatty acid composition of BSFL, myristic acid (C14:0) and palmitic acid (C16:0) comprised the largest proportion of saturated fatty acids. In larvae that are fed coconut press cake, the rate of C14:0 abandonment is significantly higher than that of another diet. This finding is consistent with the findings of Li et al. (34), who reported that the C14:0 fatty acid content of BSFL fed a coconut oil diet was substantially higher. Coconut oil is present in coconut press cake in the form of ether extract at an approximate concentration of 38.08% (35, 36). The reduction in MUFAs was primarily due to a decrease in oleic acid (C18:1 n9c). BSFL that were fed soy milk residue exhibited the greatest levels of C18:1 n9c and linoleic acid (C18:2n6c), as demonstrated by this result. Dietary linoleic acid demonstrates a beneficial relationship with palmitic and stearic acids in BSFL. Li et al. (34) demonstrated that BSFL generated specific amounts of palmitic acid (C16:0) and stearic acid (C18:0) from acetyl-CoA via malonyl-CoA synthesis. Linoleic acid undergoes β-oxidation to produce acetyl-CoA, which is then transformed into malonyl-CoA and employed in *de novo* fatty acid synthesis. Palmitic acid (C16:0) and stearic acid (C18:0) are synthesized through the activity of fatty acid synthase and elongase enzymes. This outcome reveals that the ingestion of soy milk residues enhances the concentration of unsaturated fatty acids in BSFL (26, 37, 38).

## 5 Conclusions

The BSF is regarded as one of the most promising options in insect farming for feed and food because of its physiological traits and superior nutritional qualities. This study highlights the potential of using organic by-products as substrates for rearing BSFL, which can produce highly nutritious feed. The evaluation of BSFL reared on various substrates—chicken feed, pig feed, soy milk residue, coconut press cake, and perilla cake—revealed significant variations in their nutritional profiles. The larvae exhibited high CP levels, particularly when fed chicken feed, pig feed, soy milk residue, and perilla cake, indicating their suitability for protein-rich feed applications. Soy milk residue and perilla cake provided the highest crude fiber content, enhancing the fiber profile of the larvae. Additionally, favorable growth metrics of the larvae, including weight, length, and width, demonstrate the effectiveness of these substrates in supporting larval development. The fatty acid profile of the larvae, with notable amounts of various essential fatty acids, further underscores the nutritional value of BSFL. Finally, this study demonstrates that by providing BSFL with substrates based on a wide range of various combinations of plant by-products, it is possible to achieve protein quality in BSF prepupae that is very close to that produced with commercial animal feed. However, when the meal has very low-quality nutritional content, the development of BSF biomass is less efficient, resulting in a reduced amount of protein produced. Thus, when using these substrates, the benefit of reusing leftovers should be carefully balanced against the disadvantage of having a somewhat smaller amount of protein. Overall, these findings are particularly important in view of promoting BSF as a flexible tool that may bio-convert a wide range of plant by-products to high-quality nutritious content and suggest that utilizing organic by-products in Thailand for BSFL production is a viable strategy for creating nutrient-dense feed, contributing to sustainable waste management and enhancing feed quality in livestock production.

## Data Availability

The raw data supporting the conclusions of this article will be made available by the authors, without undue reservation.
