# Correction: Evaluating the nutrient and fatty acid profiles of black soldier fly larvae (*Hermetia illucens*) raised on various diets in Thailand

**DOI:** 10.3389/finsc.2026.1794283

**Published:** 2026-02-12

**Authors:** Sarayut Pittarate, Chaiwat Arjin, Perumal Vivekanandhan, Kannan Swathy, Chun-I Chiu, Supamit Mekchay, Patipan Hnokaew, Apinya Sartsook, Thanandon Siripan, Korawan Sringarm, Patcharin Krutmuang

**Affiliations:** 1 Office of Research Administration, Chiang Mai University, Chiang Mai, Thailand; 2 Department of Entomology and Plant Pathology, Faculty of Agriculture, Chiang Mai University, Chiang Mai, Thailand; 3 Center of Excellence on Agricultural Biotechnology: (AG-BIO/MHESI), Bangkok, Thailand; 4 Center of Omics for High-Value Agriculture (AgOmics-CMU), Faculty of Agriculture, Chiang Mai University, Chiang Mai, Thailand; 5 Department of Animal and Aquatic Sciences, Faculty of Agriculture, Chiang Mai University, Chiang Mai, Thailand

**Keywords:** black soldier fly larvae, fatty acid profile, nutritional value, *Hermetia illucens*, zero hunger, responsible consumption and production, life on land

There was a mistake in the caption of **Figure 1** as published. This read: “The Pearson correlation between dietary chemical composition and BSFL growth performance.”

The correct caption of Figure 1 appears below:

“The Pearson correlation between dietary chemical composition and BSFL growth performance, An asterisk indicates a statistically significant difference (^*^p < 0.05).”

There was a mistake in the caption of **Figure 3** as published. This read: “Principal component analysis (PCA) biplots were used to illustrate the correlation between the fatty acids in larvae and their dietary type. In the *X-* axis, PC1 accounted for 76.09% of the total variability, whereas PC2 accounted for 17.86% on the *Y*-axis.”

The corrected caption of Figure 3 appears below:

“Principal component analysis (PCA) biplots were used to illustrate the correlation between the fatty acids in larvae and their dietary type. In the *X-* axis, PC1 accounted for 75.93% of the total variability, whereas PC2 accounted for 18.01% on the *Y*-axis.”

There was a mistake in the caption of **Figure 4** as published. This read: “The Pearson correlation between the fatty acid composition of the diet and that of the BSFL.”

The corrected caption of Figure 4 appears below:

“The Pearson correlation between the fatty acid composition of the diet and that of the BSFL, Asterisks indicate statistically significant differences (^*^p < 0.05, ^**^p < 0.01).”

There was a mistake in **Table 1** as published. In row 5, “Ether extract”, the letters of difference “3.44 ± 0.54^b^, 9.50 ± 0.03^c^, 10.20 ± 1.20^c^, 27.28 ± 0.12^d^, 10.52 ± 0.04^b^” should read: “3.44 ± 0.54^a^, 9.50 ± 0.03^b^, 10.20 ± 1.20^b^, 27.28 ± 0.12^c^, 10.52 ± 0.04^b^”

There was a mistake in **Table 3** as published. “Feed cost/TH bath*” should read “Feed cost, THB^†^”. The footnote, “* Cost of feed per 100 g of larva,” should read, “^†^THB, Thai Baht; Feed cost was calculated per 100 g of larva,”.

In the **Abstract**, the **Results** section originally contained the sentence, “Favorable growth performance was observed, with larvae reaching 0.22 ± 0.01 g in weight, 20.38 ± 0.36 mm in length, and 5.08 ± 0.05 mm in width.” This has been deleted so that the section now reads:

“**Results**: Significant differences in nutritional composition were observed among substrates. Crude protein content was high in larvae fed chicken feed (50.55 ± 0.07%), pig feed (52.10 ± 0.14%), soy milk residue (52.15 ± 0.78%), and perilla cake (47.20 ± 0.00%). Crude fiber was highest in larvae fed soy milk residue (7.19 ± 1.48%) and perilla cake (5.38 ± 0.25%). Fatty acid analysis revealed substantial levels of saturated and unsaturated fatty acids, including palmitic, oleic, and linoleic acids. Larvae reared on coconut press cake showed the highest saturated fatty acid content (74.91 ± 0.03%), while those fed soy milk residue exhibited the highest oleic (26.68 ± 0.06%) and linoleic acid (38.44 ± 0.07%) contents, resulting in increased polyunsaturated fatty acids (38.57 ± 0.03%).”

In Section 3.2, “*Chemical composition of the BSFL after feeding diets*”, the sentence “The DM and crude fiber of all diets show no significant differences (*p* < 0.005).” has been corrected to read:

“The DM and crude fiber of all diets show no significant differences (*p* > 0.05).”

In the same section, the sentence “We found that the CP and ether extract show the highest percentage from larvae fed on chicken feed and coconut press cake, respectively.” has been corrected to read:

“We found that the CP and ether extract show the highest percentage from larvae fed on soy milk residue and coconut press cake, respectively.”

Also in the same section, the sentence “Larvae reared on coconut press cake contained a higher proportion of ether extract but lower CP content, whereas those fed chicken feed exhibited the greatest pupal weight, length, and width (*p* < 0.05).” has been corrected to read:

“Larvae reared on coconut press cake contained a higher proportion of ether extract but lower CP content, whereas those fed chicken feed exhibited the greatest pupal weight, length, and width (data not shown, *p* < 0.05).”

In Section 3.3, “*Fatty acid profiles in BSFL*”, the sentence “Dietary sources contributed substantially to the variation in fatty acid profiles, as evidenced by the first two principal components (PC1 = 75.93% and PC2 = 18.01%), which together explained 93.95% of the total variance.” has been corrected to read:

“Dietary sources contributed substantially to the variation in fatty acid profiles, as evidenced by the first two principal components (PC1 = 75.93% and PC2 = 18.01%), which together explained 93.94% of the total variance.”

The original version of this article has been updated.

